# Textile Supplies for Hospitals

**Published:** 1916-05-13

**Authors:** 


### TEXTILE SUPPLIES FOR HOSPITALS.

### XI.—Surgical Dressings.

The name of cotton-wool in its earlier connotations denoted simply the raw cotton of commerce, the fruit of what Herodotus called the wool-bearing trees. The absorbent cotton-wool known to surgery within the last forty years or so has, upon the other hand, been a somewhat elaborately prepared or manufactured article. The basis is raw cotton, and a properly prepared absorbent wool presents cotton in its purest form, deprived of the organic matters which might putrefy, and which certainly stand in the way of the greedy absorbency manifested by cotton when it has been reduced to a pure cellulose.

The cotton used for dressings is not that which is imported for cotton spinners, although eventually some of that cotton is consumed by makers of waddings. "Linters" are used, or some form of what is called cotton-waste. In other words, the material taken is a by-product of the regular cotton manufacturing industry. The linters are such lint or fibres of cotton as remain adherent to the cotton-seed after it has passed through the cotton-gin by which the main part of the fibre is removed; the material is virgin cotton, deteriorated for the regular purposes of industry by the severity of the mechanical process of recovering it from the seed. The cotton-waste is that known technically as card strips, card fly, and comber waste. The names are less illuminative than could be desired, but they describe by-products of the cotton mill. Raw cotton is treated on carding machines to tease out its fibres into a delicate web, and in course of that operation some broken fibre, less suitable for yarn-spinning, is removed or stripped, and designated "strips." Equally in carding, a certain proportion of fibre floats in the air, and eventually settles, and is swept up and called "fly." The comber waste arises from the systematic abstraction of the shorter fibre out of long-stapled cottons, like the Egyptian varieties. A material neither too long nor too short in staple, clean, ripe, and of good colour is to be preferred. Qualities vary in absorbent wool as they do in other forms of manufactured cotton, and the less desirable and cheapest are those made from cotton-waste. Linters and comber waste provide the best qualities, and classes of inferior cotton-waste too poor for surgical use are made into ordinary cotton wadding, or converted into gun-cotton, imitation silk, electric-lamp filaments, collodion, varnish, and other remotely related articles. These unspun cotton-wastes are distinct, of course, from the thread-wastes used by engineers in cleaning oily machinery.

### Absorbent Cotton-Wool.

The manufacturing treatment is twofold; mechanical for the removal of loose dirt and the stroking-out of the fibres, and chemical for the extraction of oil and the natural impurities present in cotton. The treatment begins normally with a process for beating and shaking out foreign matter. The chemical operations follow, and, except that they are more severe, the processes are those of ordinary bleaching. The material is boiled for from six to ten hours in a 2 or 3 per cent. solution of caustic soda and soda ash under a pressure of four atmospheres. The cotton is washed thoroughly, and for another one or two hours is penetrated by a circulating stream of weak hydrochloric acid. For a further three or four hours it is treated with chlorine derived from weak hypochlorite of soda. After another washing the material is neutralised in sulphurous acid and washed with copious supplies of water. The methods pursued vary in detail, particularly as to the strengths of the reagents and the length of time in boiling, but the broad effect is the same. The treatment is made as severe as the material will bear, with the object of producing a fully bleached and pure cellulose. Superfluous treatments are sometimes added, and to heighten the apparent whiteness the cotton is often blued before being dried and passing on to the purely mechanical processes.

To lend the crispness of touch that is technically called "scroop" the bleached cotton is treated sometimes with dilute tartaric acid and soap. The features of blue-whiteness and crispness are presumably demanded or they would not be cultivated; but it does not appear that they add anything to the real utility of the material, and there is even a possibility of their detracting from it. The drastic bleaching reduces the weight of the cotton, and there is some temptation to add to it glycerine, soap, or deliquescents like calcium chloride, which help to restore the lost weight and to improve the apparent absorbency without making the material actually more desirable in use.

### Carding.

The cotton comes away from bleaching in a somewhat matted state, and the first process to which it is subjected aims at opening it into a free condition by the passage through it of spikes carried upon a rotating drum. The material is then ready to be acted upon by the more delicate wire teeth of the carding engine which coax out the fibres into a filmy veil. This web, in leaving the machine, is wound around a wooden cylinder until a sufficient number of layers have been coiled. When this fleece is cut from the drum it exists in the form of a blanket of loose felt, seven or eight feet long and half as many feet wide. In this state the wool lends itself to impregnation with medicaments, and all that remains to be done is to roll up the fleece on a simple winding machine, interleaving the folds with paper if desired. The product is a 4-foot roll of wadding of homogeneous density, and its division into portions of a determined weight is effected by a mechanical knife

Note.—Previous articles in this series appeared on December 18, 1915, and January 8 and 22, February 5 and 19, March 4, 18, and April 1, 15, 29, 1916. The next article will treat of "The Purchasing of Stores."

It has been computed that in one pound of absorbent cotton-wool there are some 150,000,000 individual fibres, and in a good sample the fibres are straight and fairly even in length. In the lower qualities there is a noticeable presence of "neps"—to use the trade name for the little buttons of curled fibres produced by defects in carding, which are conspicuously plentiful in wadding produced from card strips. A tuft of the cotton thrown on the surface of cold water should sink at once, and it should sink quite as quickly after having been washed, wrung, and dried. A moistened sample should be without reaction either upon red or blue litmus paper. And when burned the residue of ash should be slight; ash not exceeding 1 per cent. is prescribed by the British Pharmacopœia, but an allowance not exceeding 0.3 per cent. is the German standard, which might well be copied in this country.

### Gauze and Lint.

Cotton-wool is of high absorptive power relatively to all other loose dressings, but it is necessarily inferior in capillarity to fabrics in which the fibres have been joined by spinning. Partly upon this account wool is extensively used in conjunction with woven gauze. The fabric is strictly not a gauze at all, for in true gauze pairs of adjoining warp threads are crossed upon each other by a special arrangement in weaving. The surgeon's gauze is a muslin of extraordinarily open texture woven from fine yarn, soft to the touch, necessitating the use of a relatively high quality of raw cotton. In the nature of the case gauze is very much more expensive than wool, taking weight as the basis of comparison, although the disparity is offset in some degree by the greater capillarity. Gauze is often used to encage cotton-wool, and in serving to hold the loose mass together it assists usefully in absorbing the discharge from wounds.

Surgical lint in some degree combines the two characters of gauze and wool. It is a fabric woven with a warp made from fibre not significantly different from that which is used for absorbent cotton-wool and a weft corresponding closely to that employed in gauze. The cloth is bleached to obtain as great absorptive value as possible, and the continuity of the structure favours capillary power. This continuity is in part undone by the measures taken to create a fluffy surface. The face of the cloth is scratched by wire-toothed rollers alternately revolving in opposed directions so that fibre is gradually disengaged from the soft spun warp and the thread is more or less completely disintegrated. There remains, however, a coherency of structure that is absent from wads of cotton-wool, and by grace of the raised pile the fabric gives protection against the chilling of the wound.

### Oiled Silk.

The oiled silk used to check evaporation above a dressing of cotton is an article over which many pains are expended. The best procurable Piedmont raw silks are selected in Italy and are "thrown" in English factories. Care is exerted to remove all rough and uneven portions, and the yarn is woven into a light, open muslin cloth. Silk of such quality is expensive, but it is brought within practical reach by the compensating factor which allows many yards of the fabric to be woven from a single pound. The fabric is coated with successive layers of drying oil, of which linseed oil is the best, and this oxydises to form a resinous coating impervious to moisture. A cotton substructure might serve, but the fatty acids present in the drying oils are detrimental in time to cotton; an oiled cotton may be unpleasantly combustible, and has been known to become a spontaneous source of fire. Silk is the more flexible and secure foundation, and as cotton equally fine would be almost equally costly and less satisfactory its employment is fully justified.

Cotton cloths variously described as jaconette or batiste are treated with rubber or gutta-percha to make waterproof coverings for dressings, and they are simply varieties of plain cotton cloth. These coverings are near relations of the waterproof sheetings that are made by spreading upon a base of calico a mixture of rubber, sulphur, and mineral matter mixed with a spirit solvent and finally cured by steam heat. The merit of these sheetings resides more in the coating than the fabric. The materials employed are of secondary consequence to the proportions of their employment, the temperatures used, and to a number of small details as to which manufacturers preserve silence jealously. The direction is one in which new progress is rather more possible than in the case of the purely fibrous materials. The progress made in late years in coating cottons with changed forms of cellulose suggests that further possibilities are in store and that the collaboration of chemists with manufacturers may produce alternatives to the use of oil and rubber for waterproof coatings.

### Elastic Bandages.

Rubber holds its own place in the ordinary elastic web bandage made by combining square-cut threads of pure rubber in a silk or cotton fabric. Of late years an elastic bandage made without rubber has been placed on the market, and its features are not without interest. As anybody may verify with the aid of his fingers and a short length of string, a thread twisted beyond a certain degree tends to contract and to double back upon itself. Advantage is taken of this feature in weaving heavily twisted yarn in a loom which permits of a high tension. The fabric is woven in open texture, and on the release of the tension the force of the twist exerts itself to close the interstices. To prevent the whole fabric from bending over in the direction of the twist the bandages are woven with threads twisted in right- and left-hand spirals alternately. Thus the symmetry of the structure is preserved, and at the same time a steady elastic pressure is exerted upon the limb around which the bandage is stretched. The bandages are made both in worsted and cotton, and although necessarily more expensive than the ordinary non-elastic article they are appreciably cheaper than elastic web.

